# Optimal timing for triggering oocyte maturation during in vitro fertilization cycles varies between gonadotropin-releasing hormone agonist and human chorionic gonadotropin use

**DOI:** 10.1016/j.xfre.2025.07.009

**Published:** 2025-07-29

**Authors:** Noritoshi Enatsu, Kohyu Furuhashi, Junko Otsuki, Kunihiro Enatsu, Eri Okamoto, Shoji Kokeguchi, Masahide Shiotani

**Affiliations:** Hanabusa Women’s Clinic, Kobe City, Hyogo, Japan

**Keywords:** Triggering oocyte maturation, hCG trigger, GnRH agonist trigger, in vitro fertilization (IVF)

## Abstract

**Objective:**

To investigate the optimal timing for triggering oocyte maturation by evaluating the interval from the trigger to oocyte retrieval and the subsequent in vitro fertilization outcomes. The administration of human chorionic gonadotropin (hCG) or gonadotropin-releasing hormone agonist (GnRHa) is a critical trigger for the final maturation of oocytes within in vitro fertilization cycles. The interval between the trigger and oocyte retrieval has typically been set at 34–40 hours. However, the best timing on the basis of the type of trigger is still unclear.

**Design:**

A single-center retrospective cohort study.

**Subjects:**

A total of 59,206 oocyte retrieval cycles were investigated at our clinic between April 2010 and March 2024. Cycles with luteinizing hormone surge and those undergoing oocyte retrieval on the day after the trigger were excluded.

**Exposure:**

The interval from trigger to oocyte retrieval was from GnRHa or hCG administration to oocyte retrieval initiation. The subjects were categorized into the following two groups according to the type of oocyte maturation trigger used: GnRHa group (buserelin [600 μg] nasal spray) and hCG group (hCG [3,000–10,000 IU] or choriogonadotropin alfa [250 μg] subcutaneously).

**Main Outcome Measures:**

The primary outcome measure was the total number of mature metaphase II (MII) oocytes retrieved.

**Results:**

The GnRHa trigger group had a higher number of MII oocytes retrieved with a longer interval than with a shorter interval (7.2 ± 6.5 vs. 4.3 ± 5.3), whereas the hCG trigger group had fewer oocytes with a longer interval than with a shorter interval (4.0 ± 4.6 vs. 6.9 ± 5.8). The differences between shorter and longer intervals became more pronounced in older age groups. Although the MII ratio per oocyte did not show significant difference with the interval duration, the blastocyst formation rate and total number of blastocysts were markedly higher when the interval was longer than 36.5 hours in the GnRHa group.

**Conclusion:**

The optimal interval varies between the GnRHa trigger and the hCG trigger, with the hCG trigger associated with a shorter interval for obtaining the maximum number of MII oocytes than the GnRHa trigger.

In the natural oocyte development cycle in women, luteinizing hormone (LH) surge plays a critical role in resuming meiosis, which is paused at the immature metaphase I stage, and advancing it to the mature metaphase II (MII) stage (1, 2). In in vitro fertilization (IVF) process, a similar progression of meiosis is necessary before oocyte retrieval. During oocyte maturation, the first polar body is extruded, allowing the diploid cell to transition into a haploid gamete capable of being fertilized by a spermatozoon (3). Hence, the gonadotropin-releasing hormone (GnRH) agonist (GnRHa) and human chorionic gonadotropin (hCG) are widely employed as the “trigger” of oocyte maturation in IVF cycles. Gonadotropin-releasing hormone agonist induces the endogenous LH surge and promotes oocyte maturation. Meanwhile, hCG does not increase the LH levels; however, it has a molecular structure similar to that of LH, thereby exerting comparable physiological effects (4). It induces a biologic response analogous to the LH surge, facilitating the final maturation of oocytes. Consequently, hCG is widely used as a maturation inducer in IVF procedures.

In most IVF cycles, the commonly practiced interval is 32–36 hours. Bjercke et al. (5) reported that administering hCG 34–38 hours before oocyte retrieval does not affect IVF outcomes. However, a meta-analysis revealed that the percentage of mature MII oocytes can be increased by prolonging the interval between the hCG trigger and oocyte retrieval (6). More recently, in the analysis of cases where preimplantation genetic testing was performed at an hCG-oocyte retrieval interval of 34–39 hours, the longer interval resulted in higher probability of euploidy for blastocysts (7).

These studies indicate that the time interval between the trigger and oocyte retrieval is a critical factor influencing oocyte retrieval outcomes. However, until recently, most of these analyses concentrated on results derived from the hCG trigger, whereas available large-scale data on the GnRHa trigger remain limited. Owing to the shorter duration of action of GnRHa than that of hCG, GnRHa is considered less suitable for sustaining luteal function when used as a trigger, potentially resulting in suboptimal outcomes in fresh embryo transfers (8). However, this shorter duration serves a significant advantage in mitigating ovarian hyperstimulation syndrome risk, particularly in younger patients with robust ovarian function. Consequently, the combination of the GnRHa trigger and the freeze-all strategy has become common in many countries (9, 10, 11, 12). However, a comprehensive evaluation of the GnRHa trigger remains inadequate.

Therefore, this retrospective analysis meticulously examined the effects of the interval from the trigger to oocyte retrieval on IVF outcomes for the GnRHa and hCG triggers in a large number of patients.

## Materials and methods

### Patient population

This retrospective analysis encompassed 69,819 IVF cycles conducted at the private clinic between April 2010 and March 2024, focusing on cases involving oocyte retrieval. Exclusions were made for cycles involving patients aged ≥45 years, oocyte cryopreservation, or oocyte donation. Additionally, cycles using testicular sperm extraction or in vitro maturation that may impact fertilization or blastocyst development were excluded. The interval between the oocyte maturation trigger and retrieval was meticulously calculated on the basis of the timing of trigger administration (GnRHa or hCG) and the initiation of oocyte retrieval procedures. A total of 7,182 cycles were omitted because of retrieval occurring the day after the trigger, as a result of either an LH surge on the trigger date or other irregular circumstances. Additionally, 3,431 cycles that used dual triggers—combining GnRH and hCG—were excluded because dual trigger was used specifically to cases where the initial oocyte retrieval produced fewer mature oocytes than expected, indicating a less optimal response to the trigger. Consequently, the patients’ backgrounds and assisted reproductive technology (ART) histories differed in dual trigger cases, making direct comparisons with other groups unsuitable for this study. Ultimately, 59,206 cycles were analyzed.

### IVF cycle management

The stimulation protocols had no restriction, and the standard controlled ovarian hyperstimulation protocol was used. For oocyte stimulation, oral ovulation induction agents (letrozole, clomiphene citrate, or cyclofenil), human menopausal gonadotropin, and recombinant follicle-stimulating hormone were employed, either alone or in combination. To prevent premature LH surges, GnRHa, GnRH antagonist, or oral progesterone was used. The final oocyte maturation trigger was administered when at least two follicles reached a diameter of 17–18 mm or when the attending physician determined the follicular cohort was mature.

Gonadotropin-releasing hormone agonist was used as a trigger in cycles wherein GnRHa was not employed for ovulation suppression during controlled ovarian hyperstimulation, such as in GnRH antagonist protocols and progestin-primed ovarian stimulation protocols. In cycles wherein GnRHa was used for ovulation suppression, such as in long or short agonist protocols, as well as in mild or antagonist cycle planning for fresh embryo transfer, hCG was used for final maturation trigger.

The intended interval between the trigger and oocyte retrieval was set at 36 hours. However, deviations occasionally occurred for various reasons. For instance, if a patient’s visit on the day of the trigger was in the evening, later than the scheduled trigger time, the interval would be shortened. Conversely, if the trigger proceeded as planned but the oocyte retrieval procedure was delayed because of other patients’ procedures and operating room preparation, the interval would be extended. Additionally, there were occasional instances of patient errors associated with staggered intervals. Consequently, discrepancies of up to approximately 4 hours were observed among patients. Both the exact timing of the trigger administration and the initiation of the retrieval procedure were meticulously recorded to ensure accurate calculation of the interval preceding oocyte retrieval.

### Classification according to the trigger method

We categorized the cases into two groups according to the type of trigger used: the GnRHa and hCG groups. In the GnRHa group, a 600-μg dose of buserelin nasal spray was administered as the trigger, followed by an additional 600-μg dose 1 hour after the initial administration to reduce the risk of suboptimal oocyte retrieval (13). The time of the initial buserelin administration was defined as the trigger time. In the hCG group, either 3,000–10,000 IU of hCG or 250 μg of choriogonadotropin alfa was subcutaneously injected, depending on the patient’s body habitus.

### Laboratory intervention

After oocyte retrieval, we conducted cumulus stripping and calculated the MII ratio (MII ratio = the number of MII oocytes/total number of oocytes). In vitro fertilization or intracytoplasmic sperm injection was conducted according to the semen analysis results and prior fertilization outcomes. The embryos were cultured to the cleavage or blastocyst stage and then either transferred or cryopreserved. Blastocysts were classified according to the Gardner grading scale (14). Those with grade 3BB or better on day 5 defined high-quality blastocysts. In calculating the blastocyst rate and the high-quality blastocyst rate, we excluded embryos undergoing cleavage-stage embryo transfer or freezing and used the number of embryos intended for blastocyst culture as the denominator.

### Statistical analysis

The primary outcome measured was the total number of mature (MII) oocytes retrieved, and the secondary outcomes included the number of blastocysts and high-quality blastocysts. Initially, scatterplots were used to identify trends for subsequent statistical analyses, specifically examining the relationship between the interval from trigger administration to oocyte retrieval and the number of MII oocytes retrieved. The results were organized on the basis of time intervals, with the mean number of oocytes retrieved per 100 cases plotted to discern trends. Additionally, the data were stratified by the type of trigger used and analyzed for trends across different age groups. On the basis of scatterplot, 36.5 hours of interval was established as a reference point for analysis.

Statistical analyses were conducted between groups with a time interval over 36.5 hours and those with a time interval under 36.5 hours. As statistical analysis methods, the Welch t-test was used to compare the means of continuous variables between two groups, and the categorical variables were compared using the chi-square test and are reported as proportions. Results of continuous variables are reported as the means ± SDs and *P* value, and results of categorical variables are written in percentages and risks ratio with 99% confidence intervals (CIs). Results with a *P* value of <.01 or a 99% CI excluding 1.0 are the difference considered as statistically significant. Risk ratios provide additional context to in proportion. A value higher than 1.0 indicates that shorter intervals are associated with larger proportions, and a higher deviation from 1.0 suggests a trend toward more significant differences. In addition to *P* value calculated by t-test, the Cohen D was used to measure the effect size between two groups. This metric provides a quantitative evaluation of the magnitude of differences, which may not be evident through statistically significant differences (*P* values). According to the Cohen D, a value of 0.2–0.5 indicates a small difference, 0.5–0.8 suggests a moderate difference, and ≥0.8 represents a large difference. Furthermore, multiple linear regression analyses were conducted to adjust for age and other confounding factors affecting ovarian response. Age, serum antimüllerian hormone (AMH) levels, and the number of ART cycles (including the current cycle) were identified as confounders and analyzed alongside the interval.

All statistical data were analyzed using Excel (Microsoft 365) and EZR (Saitama Medical Center, Jichi Medical University, Saitama, Japan), which is a graphical user interface for R (The R Foundation for Statistical Computing, Vienna, Austria).

### Ethical considerations

This study is a retrospective cohort analysis, and no interventions were made regarding the IVF treatment process. The Institutional Review Board of Hanabusa Women’s Clinic, which includes members selected by the institution and an external third-party institution, approved this study (approval number, 2024-05). Patients provided informed consent before the treatment period preceding IVF cycles, and separate confirmation for data analysis and publication via anonymization was obtained independently of treatment consent. Only patients who consented to provide their data were included in the database.

## Results

The statistical analysis of oocyte retrieval cycles yielded the following results. The GnRHa group and hCG trigger groups included 40,793 and 18,413 cycles, respectively. The mean intervals between the administration of the maturation trigger and oocyte retrieval were 36.4 ± 0.9 hours in the GnRHa group and 36.4 ± 1.0 hours in the hCG group. Table 1 shows the baseline characteristics of patients categorized into 4 groups on the basis of the type of trigger used (GnRHa trigger or hCG trigger) and the interval between maturation trigger and oocyte retrieval (interval of <36.5 hours or interval of ≥36.5 hours). In the GnRHa groups, patients with the interval of ≥36.5 hours were younger than those in the <36.5 hours (39.1 ± 4.6 and 38.0 ± 4.8, respectively; *P*<.01; Cohen D, 0.23). Statistical analysis identified differences in AMH, the number of ART attempts, and LH at trigger via the t-test, attributable to the large sample size. However, the value of the Cohen D was less than 0.2, indicating that the effects of differences between the 2 groups were minimal. In the hCG group, the Cohen D values were below 0.2 for all variables, indicating that differences between intervals of <36.5 hours and those of ≥36.5 hours are minimal.

**Table 1:** The baseline characteristics of patients categorized on the basis of the type of trigger used.

	Mean ± SD	Mean ± SD	*P* value	Cohen D
GnRHa trigger	Interval of <36.5 h (n = 25,572)	Interval of ≥36.5 h (n = 15,221)		
Age	39.1 ± 4.6	38.0 ± 4.8	<.01	0.23
AMH (ng/mL)	2.2 ± 2.6	2.1 ± 2.9	<.01	0.04
No. of ART attempt	4.6 ± 3.9	4.9 ± 3.2	<.01	0.12
Interval (hours)	35.7 ± 1.4	37.2 ± 0.7	<.01	0.41
Basal FSH (mIU/mL)	6.0 ± 3.1	6.0 ± 4.9	.72	0.01
LH at trigger (mIU/mL)	4.7 ± 4.9	4.3 ± 4.3	<.01	0.09
hCG trigger	Interval of <36.5 h (n = 9,428)	Interval of ≥36.5 h (n = 8,985)		
Age	39.4 ± 4.5	39.1 ± 4.2	<.01	0.06
AMH (ng/mL)	1.6 ± 2.0	1.6 ± 1.6	.80	0.01
No. of ART attempt	4.9 ± 3.2	4.7 ± 3.3	<.01	0.06
Interval (h)	35.8 ± 1.7	37.4 ± 0.8	<.01	0.33
Basal FSH (mIU/mL)	6.8 ± 6.4	7.3 ± 3.9	<.01	0.06
LH at trigger (mIU/mL)	5.3 ± 4.5	5.5 ± 3.5	<.01	0.05

*Note:* The table includes essential parameters such as age, AMH levels, number of ART attempt (including current cycle), basal FSH levels, and LH levels at trigger. Each parameter is presented with mean values ± SDs for each group, along with their statistical significance indicated by *P* values. *P* values were calculated using the Welch t-test, along with the effect size (Cohen D). The interpretation of the Cohen D effect sizes indicated that a value of 0.2–0.5 represented a small difference, 0.5–0.8 represented a moderate difference, and ≥0.8 represented a large difference. AMH = antimüllerian hormone; ART = assisted reproductive technology; FSH = follicle-stimulating hormone; LH = luteinizing hormone.

**Figure 1:**
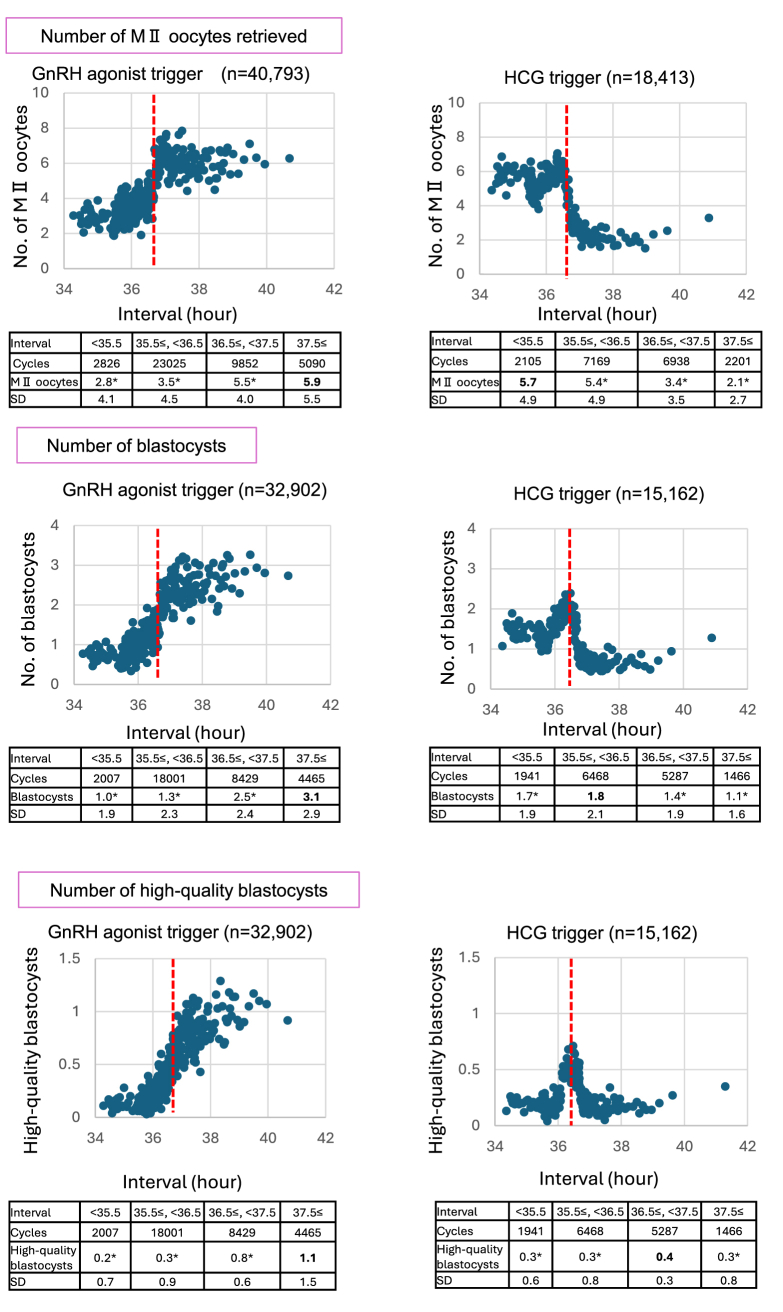
Correlation between the interval from trigger to oocyte retrieval and the oocyte retrieval outcomes (including the mean numbers of oocytes retrieved, blastocysts, and high-quality blastocysts). Each data point represents the mean values for every 100 cases, starting from shorter intervals. The red broken line indicates the reference point where the interval from the trigger to retrieval was 36.5 hours. The table below presents the outcomes, segmented by hourly intervals. In the hourly comparison, the section with the highest count is highlighted in bold. An asterisk (∗) indicates that a Welch t-test showed *P*<.01 when compared with the section with highest count. GnRH = gonadotropin-releasing hormone; hCG = human chorionic gonadotropin; MII = metaphase II.

Figure 1 illustrates the relationship between the interval from trigger administration to oocyte retrieval and the outcomes, including the number of MII oocytes retrieved, blastocysts, and high-quality blastocysts. The scatterplot highlights the trends in both the GnRHa and hCG groups, with noticeable changes observed around the 36.5 hours mark. Consequently, a reference point of 36.5 hours was chosen for analysis, with hourly comparisons conducted between the section exhibiting the highest count and other sections. Hourly comparisons were made between the section with the highest count and other sections. Among the GnRHa group, the number of MII oocytes retrieved was significantly higher in groups with an interval of >37.5 hours (mean, 5.9; SD, 5.5) than with other intervals (*P*<.01). Similarly, the number of blastocysts and high-quality blastocysts was significantly higher in the GnRHa trigger group with an interval exceeding 37.5 hours than with other intervals (3.1 and 1.1, respectively; *P*<.01). Conversely, the hCG trigger group demonstrated a significantly higher number of MII oocytes in the group with an interval shorter than 35.5 hours (mean, 5.7; SD, 4.9) than with other intervals (*P* < .01). The intervals for the largest number of blastocysts and high-quality blastocysts varied, with the highest numbers observed at 35.5–36.5 and 36.5–37.5 hours (1.8 and 0.4, respectively; *P* < .01).

Table 2 presents a comparison of oocyte retrieval outcomes on the basis of intervals shorter or longer than 36.5 hours, encompassing all age groups as well as subanalyses categorized by three specific age ranges: <35; 35–40; and >40 years. In all age groups, the GnRHa trigger group had a higher number of MII oocytes retrieved with a longer interval than with a shorter interval (7.2 ± 6.5 vs. 4.3 ± 5.3, *P* < .01), whereas the hCG trigger group had fewer oocytes with a longer interval than with a shorter interval (4.0 ± 4.6 vs. 6.9 ± 5.8, *P* < .01). According to the Cohen D, the differences between shorter and longer intervals were more pronounced in older age groups for both the GnRHa group (age < 35 years, 0.27; 35 ≤ age < 40 years, 0.35; and 40 ≤ age < 45 years, 0.41) and the hCG group (age < 35 years, 0.36; 35 ≤ age < 40 years, 0.52; and 40 ≤ age < 45 years, 0.53).

**Table 2:** The relationship between the interval from trigger to oocyte retrieval (<36.5 vs. ≥36.5 hours) and oocyte retrieval outcomes.

Interval between trigger and oocyte retrieval (h)	GnRHa trigger (n = 40,793)			hCG trigger (n = 18,413)		
	<36.5	≥36.5	*P* value (Cohen D)/risk ratio (99% CI)	<36.5	≥36.5	*P* value (Cohen D)/risk ratio (99% CI)
Overall						
No. of cycles	25,572	15,221		9,428	8,985	
No. of oocytes per cycle	**4.3** ± **5.3**	**7.2** ± **6.5**	**<0.01 (0.40)**	**6.9** ± **5.8**	**4.0** ± **4.6**	**<0.01 (0.55)**
No. of MII oocyte per cycle	**3.5** ± **4.6**	**5.8** ± **5.7**	**<0.01 (0.37)**	**5.5** ± **4.8**	**3.2** ± **4.1**	**<0.01 (0.49)**
MII ratio/oocyte (%)	81.3	80.2	1.0 (0.9–1.1)	80.4	79.8	1.0 (0.9–1.0)
Cancellation due to ovulation (%)	1.4	0.8	1.5 (0.9–8.5)	1.3	1.2	1.1 (0.8–1.5)
No. of fertilized oocytes	**2.8** ± **3.0**	**4.5** ± **4.3**	**<0.01 (0.35)**	**4.3** ± **4.3**	**2.5** ± **4.3**	**<0.01 (0.52)**
No. of blastocysts per cycle	**1.0** ± **1.8**	**2.3** ± **2.8**	**<0.01 (0.44)**	1.7 ± 1.9	1.2 ± 1.9	0.11 (0.27)
No. of high-quality blastocysts	**0.3** ± **0.8**	**0.8** ± **1.7**	**<.01 (0.38)**	0.3 ± 0.8	0.2 ± 1.0	0.78 (0.07)
Blastocyst formation rate (%)	**50.3**	**59.5**	**0.84 (0.84–0.87)**	48.8	52.2	0.94 (0.91- 1.1)
High-quality blastocyst formation rate (%)	**9.1**	**18.5**	**0.49 (0.47–0.52)**	**8.0**	**13.4**	**0.60 (0.56–0.66)**
Age of <35 y						
No. of cycles	4,626	4,657		1789	1245	
No. of oocytes per cycle	**8.8** ± **7.1**	**10.8** ± **7.2**	**<0.01 (0.27)**	**9.5** ± **7.7**	**7.2** ± **6.3**	**<0.01 (0.32)**
No. of MII oocyte per cycle	**7.2** ± **6.3**	**8.4** ± **6.5**	**<0.01 (0.18)**	**7.7** ± **5.6**	**5.7** ± **5.6**	**<0.01 (0.36)**
MII ratio/oocyte (%)	81.4	78.1	1.1 (0.9–1.2)	81.2	78.8	1.0 (0.8–1.3)
Cancellation due to ovulation (%)	0.4	0.1	4.0 (1.0–15)	0.5	1.6	0.38 (0.14–1.0)
No. of fertilized oocytes	**5.6** ± **4.9**	**6.6** ± **4.9**	**<0.01 (0.20)**	**6.0** ± **4.3**	**4.4** ± **4.3**	**<0.01 (0.37)**
No. of blastocysts per cycle	**2.4** ± **2.8**	**3.5** ± **3.3**	**<0.01 (0.36)**	2.4 ± 2.6	2.1 ± 2.6	0.56 (0.14)
No. of high-quality blastocysts	**0.6** ± **1.3**	**1.3** ± **1.7**	**<0.01 (0.46)**	0.5 ± 1.1	0.7 ± 1.4	0.11 (0.15)
Blastocyst formation rate (%)	59.3	63.2	0.94 (0.84–1.1)	55.1	58.2	0.95 (0.78–1.1)
High-quality blastocyst formation rate (%)	**13.9**	**22.9**	**0.61 (0.52–0.70)**	**11.1**	**19.1**	**0.58 (0.44–0.76)**
35 ≤ age < 40 y						
No. of cycles	7,205	5,187		3563	2596	
No. of oocytes per cycle	**5.2** ± **5.4**	**7.4** ± **6.1**	**<0.01 (0.39)**	**7.5** ± **5.4**	**4.8** ± **4.9**	**<0.01 (0.54)**
No. of MII oocyte per cycle	**4.2** ± **4.7**	**6.0** ± **5.6**	**<0.01 (0.35)**	**6.0** ± **4.8**	**3.7** ± **4.1**	**<0.01 (0.52)**
MII ratio per total number of oocyte (%)	80.8	76.8	1.1 (0.63–1.8)	80.0	76.8	1.0 (0.89–1.2)
Cancellation due to ovulation (%)	0.9	0.3	1.8 (0.98–3.3)	1.8	0.7	1.8 (0.98–3.3)
No. of fertilized oocytes	**3.3** ± **3.7**	**4.7** ± **4.2**	**<0.01 (0.34)**	**4.7** ± **3.0**	**2.8** ± **3.1**	**<0.01**
No. of blastocysts per cycle	**1.3** ± **2.2**	**2.4** ± **2.6**	**<0.01 (0.45)**	1.7 ± 1.9	1.2 ± 1.9	0.11 (0.62)
No. of high-quality blastocysts	**0.3** ± **0.9**	**0.8** ± **1.3**	**<0.01 (0.46)**	0.3 ± 0.8	0.3 ± 1.0	0.78 (0.27)
Blastocyst formation rate (%)	**57.5**	**61.1**	**0.94 (0.85–0.99)**	50.9	54.9	0.92 (0.81–1.1)
High-quality blastocyst formation rate (%)	**11.4**	**19.9**	**0.57 (0.50–0.66)**	**8.9**	**15.7**	**0.56 (0.46–0.69)**
40 ≤ age < 45 y						
No. of cycles	13,741	5,377		4076	5144	
No. of oocytes per cycle	**2.3** ± **2.8**	**3.9** ± **4.1**	**<0.01 (0.45)**	**5.2** ± **4.6**	**2.9** ± **3.4**	**<0.01 (0.58)**
No. of MII oocyte per cycle	**1.9** ± **2.5**	**3.2** ± **3.7**	**<0.01 (0.41)**	**4.2** ± **4.1**	**2.3** ± **3.0**	**<0.01 (0.53)**
MII ratio/oocyte (%)	81.6	82.5	0.99 (0.9–1.1)	80.4	81.6	0.99 (0.86–1.1)
Cancellation due to ovulation (%)	2.0	1.1	1.3 (0.9–1.9)	1.1	1.4	0.79 (0.48–1.3)
No. of fertilized oocytes	**1.6** ± **2.0**	**2.6** ± **2.9**	**<0.01 (0.41)**	**3.3** ± **3.1**	**1.9** ± **2.4**	**<0.01 (0.52)**
No. of blastocysts per cycle	**0.4** ± **1.3**	**1.1** ± **1.8**	**<0.01 (0.45)**	**1.0** ± **1.6**	**0.6** ± **1.5**	**<0.01 (0.25)**
No. of high-quality blastocysts	0.1 ± 0.4	0.3 ± 0.9	0.43 (0.30)	0.1 ± 0.6	0.1 ± 0.7	0.78 (0.00)
Blastocyst formation rate (%)	**43.5**	**54.7**	**0.80 (0.73–0.86)**	44.2	49.4	0.90 (0.80–1.0)
High-quality blastocyst formation rate (%)	**6.2**	**13.3**	**0.46 (0.40–0.53)**	**5.8**	**10.9**	**0.53 (0.43–0.66)**

*Note:* The values in the table are written in either means ± SDs for each cycle or percentages. As statistical analysis methods, the Welch t-test was used to compare the means of continuous variables between two groups, and the categorical variables were compared using the chi-square test and are reported as proportions. Results of continuous variables are reported as the means ± SDs and *P* value with the Cohen D, and results of categorical variables are written in percentages and risk ratios with 99% confidence intervals. The Cohen D showed that a value of 0.2–0.5 indicated a small difference, 0.5–0.8 suggested a moderate difference, and ≥0.8 represented a large difference. Results were considered statistically significant if the *P* value was <.01 or if the 99% confidence interval did not include 1.0, and these are highlighted in bold. Risk ratios provided additional context to the difference in proportion. A value higher than 1.0 indicated that shorter intervals were associated with larger in proportion, and a higher difference from 1.0 indicated a trend toward higher differences. The MII ratio was the number of MII oocytes/total number of oocytes. High-quality blastocysts were defined as the blastocysts with grade 3BB or better on day 5.

The MII ratio (the number of MII oocyte/total number of oocyte) was not different between the short and long intervals. In the GnRHa group, the cohort with a longer interval exhibited a higher blastocyst formation rate (59.3%) than with the shorter interval cohort (50.3%; 99% CI, 0.84–0.87). Conversely, there was no significant difference in the blastocyst formation rates between cohorts in the hCG group (48.8% vs. 52.2%; 99% CI, 0.91–1.1). Accordingly, the difference in blastocyst numbers between the short and long intervals was larger than that observed for MII oocyte numbers (Cohen D, 0.44 vs. 0.37) in the GnRHa group. Additionally, the differences were higher in the older cohort, with the Cohen D values of 0.36, 0.45, and 0.45 for age groups of <35, 35–40, and >40 years, respectively. On the other hand, the difference in the number of blastocysts in the hCG group were only revealed in the 40–45-year age group (*P*<.01; Cohen D, 0.25). Regarding high-quality blastocyst formation, a longer interval was linked to a higher rate across all subgroups. The GnRHa group had more high-quality blastocysts in the longer interval cohort than in the shorter interval cohort (0.8 ± 1.7 vs. 0.3 ± 0.8, *P*<.01). In contrast, there were no statistically significant differences in the number of high-quality blastocysts between longer and shorter intervals within the hCG groups (0.2 ± 1.0 vs. 0.3 ± 0.8, *P*=.78).

The rate of cancellation resulting from ovulation did not vary according to the interval length across any group or age category.

To control for potential confounding variables, a multiple regression analysis was conducted with the total number of MII oocytes as the dependent variable (Table 3). Upon comparison of linear, quadratic, and exponential regression models, linear regression yielded the strongest correlation (multiple R-squared, 0.4; adjusted R-squared, 0.4). Assessment of multicollinearity using variance inflation factor revealed no significant concerns, with variance inflation factor values in the GnRH group as follows: AMH, 1.3; interval, 1.1; age, 1.5; and number of ART attempts, 1.2. In the GnRHa trigger group, the regression coefficient was determined to be 2.0 (99% CI, 1.9–2.2). These findings indicate a statistically significant association between interval length (≥36.5 vs. <36.5 hours) and the total number of MII oocytes retrieved per cycle, after adjusting for age, serum AMH levels, and the number of ART cycles. In addition, a positive regression coefficient (2.0) indicates that a prolonged interval (≥36.5 hours) is associated with a significant increase in the number of MII oocytes retrieved (*P*<.01). In contrast, the hCG group exhibited a regression coefficient of −0.34, with a 99% CI of −0.19 to −0.49, indicating that a longer interval is independently associated with fewer MII oocytes retrieved.

**Table 3:** Multiple linear regression analysis predicting the total number of metaphase II oocyte retrieved.

Variable	GnRHa trigger (n = 40,793)		hCG trigger (n = 18,413)	
	Regression coefficient (99% CI)	*P* value	Regression coefficient (99% CI)	*P* value
Interval (≥36.5 h)	2.0 (1.9 to 2.2)	<.01	−0.34 (−0.49 to −0.19)	<.01
AMH (ng/mL)	0.76 (0.74 to 0.79)	<.01	1.1 (1.0 to 1.1)	<.01
Age at retrieval	−0.19 (−0.21 to −0.18)	<.01	−0.17 (−0.19 to −0.15)	<.01
No. of ART attempts	−0.07 (−0.08 to −0.06)	<.01	−0.11 (−0.12 to −0.10)	<.01

*Note:* Interval represents the duration between maturation trigger administration to oocyte retrieval initiation, divided by 36.5 hours of cut point. The number of assisted reproductive technology attempts included the current cycle. AMH = antimüllerian hormone; ART = assisted reproductive technology; CI = confidence interval; GnRHa = gonadotropin-releasing hormone agonist; hCG = human chorionic gonadotropin.

## Discussion

Through comprehensive data analysis, this study elucidates that the interval between trigger administration and oocyte retrieval significantly impacts not only the number of oocytes retrieved but also the subsequent stages of embryo development, including the blastocyst formation rates and high-quality blastocyst formation rates. According to recent meta-analyses, increasing the interval between hCG administration and oocyte retrieval markedly enhances clinical pregnancy rates (15). Similar meta-analyses have indicated that groups with intervals exceeding 36 hours had more MII oocytes than those with shorter intervals, indicating that a prolonged interval would allow for increased intrafollicular influence, enabling more in vivo maturation and subsequently, a higher yield of mature oocytes (6). However, in a single-center large cohort analysis (n = 8,811), expanding the time interval (<36 to ≥41 hours) showed no clinically relevant impact on embryological or clinical outcome (16). In the present study, the primary outcome was defined as the number of MII oocytes retrieved. This is because it has been reported that the euploidy rate is not directly correlated with the number of oocytes retrieved and the number of MII oocytes is one of the main variables affecting possibility of finding at least one euploid after adjustment for age (17). Therefore, in IVF cycles, particularly those using the freeze-all strategy combined with GnRHa trigger, retrieving an optimal number of oocytes is closely related to the success rate.

One of the pivotal findings of this study is the differences noted in the relationship between the interval and the number of oocytes retrieved in the GnRHa and hCG groups. Previous research predominantly concentrated on hCG triggers, with relatively fewer studies addressing GnRHa triggers. However, considering that the combination of the GnRHa trigger and the freeze-all strategy has become prevalent in many countries for safety reasons (9, 10, 11), re-evaluating the efficacy of the GnRHa trigger is essential. Recent studies on GnRHa triggers have compared shorter and longer intervals (<36 and ≥36 hours, respectively) and found no statistically significant difference in the number of oocytes retrieved. However, the longer interval group had slightly more oocytes than the shorter interval group (12.24 oocytes [n = 198] vs. 11.86 oocytes [n = 240]) (18). Increasing the sample size may yield results congruent with the present study findings.

This study demonstrated that, within the GnRHa trigger group, a longer interval between triggering and oocyte retrieval was associated with a higher oocyte yield. Conversely, in the hCG trigger group, a shorter interval resulted in the retrieval of more oocytes. These findings suggest that the hCG trigger initiates its effects more rapidly than the GnRHa trigger. However, studies measuring actual LH and hCG levels after a trigger demonstrated that the serum LH levels peak approximately 4 hours after GnRHa administration, whereas the serum hCG levels peak approximately 15 hours after hCG administration (19). Therefore, the observed differences may be influenced by the distinct physiological activities of hCG and LH. In fact, it is reported that hCG has a higher affinity for the LH receptor than LH and is fivefold more potent in stimulating human granulosa cell cyclic adenosine monophosphate (cAMP) activity than the equimolar concentrations of LH (20). Meiosis resumes once cAMP degradation is inhibited. Therefore, despite the slower increase in the serum hCG levels, the stronger activation of cAMP may result in an earlier resumption of meiosis through hCG triggers compared with GnRHa triggers.

Interestingly, the number of high-quality blastocysts in the hCG trigger group significantly peaked at 36.5–37.5 hours (Fig. 1). This observation can be explained by the increased high-quality blastocyst formation rate at longer intervals (13.4% vs. 8.0%; 99% CI, 0.56–0.66) (Table 2). Importantly, the hCG trigger group, similar to the GnRHa trigger group, exhibited improved outcomes at longer intervals in terms of high-quality blastocyst rates. Findings from previous studies support this trend. Wang et al. (6) demonstrated through meta-analysis that extended intervals are correlated with increased rates of MII oocytes. Additionally, Lee et al. (7) reported that a longer interval enhances euploidy rates, indicating that extended in vivo maturation periods support normal oocyte meiosis. In this study, the number of high-quality blastocysts in the hCG group peaked at 36.5–37.5 hours, which differs from the intervals yielding the highest number of MII oocytes (<35.5 hours) (Fig. 1). Consequently, it is difficult to determine the optimal interval for hCG triggers. More detailed time divisions and stratifying patient backgrounds, such as age groups and AMH levels, may assist in identifying individualized optimal intervals. Additional research is warranted.

Another important finding of this study is that the effects of intervals become more pronounced as people age. Reichman et al. (21) corroborated these findings, noting that in women aged 40 years, extended intervals (≥36.5 hours) were correlated with enhanced clinical pregnancy rates and birth rates; no notable differences were observed in younger women. It is well established that age is a crucial determinant of IVF success (22, 23). Factors such as reduced mitochondrial numbers within the oocyte cytoplasm, heightened deoxyribonucleic acid mutation rates, decreased adenosine triphosphate levels, and increased reactive oxygen species collectively diminish the developmental potential of oocytes and embryos in older patients (24). Therefore, IVF cycles for patients aged >40 years require meticulous management to optimize outcomes.

In this study, although the rate of cancellation caused by ovulation increased with age, extending the interval did not result in cancellation. Thus, extending the interval may be feasible when implementing a GnRHa trigger. In contrast, among the hCG groups, a longer interval was associated with a decrease in the number of MII oocytes retrieved. As shown in Table 2, the MII ratio remained stable regardless of whether the interval was short or long across all age categories. This finding suggests that the reduction in the total number of oocytes retrieved in the hCG group is directly related to the decreased number of MII oocytes obtained. The cause of this reduction with extended intervals in the hCG group is yet to be determined. One hypothesis posits that partial ovulation may have occurred in some patients, leading to a reduced retrieval count despite the lack of cancellation. This phenomenon appeared more pronounced within the hCG group, likely because of its stronger in vivo activity than within the GnRHa group. Additionally, it has been reported that premature luteinization may be associated with a decrease in the number of oocytes retrieved (25). Similarly, Vanni et al. (26) reported that increasing progesterone is associated with lower rates of top-quality blastocyst. The phenomenon of premature luteinization reported in these studies refers to the condition where the progesterone levels have already increased at the time of triggering. It has been noted that hCG triggers cause an increase in progesterone levels much faster than natural LH surges (27). Therefore, it is suggested that the strong in vivo activity of hCG induces early luteinization in the ovaries, leading to a decrease in the number of oocytes retrieved. Consequently, more precise time management is required when implementing an hCG trigger than when implementing a GnRHa trigger.

On the basis of the findings of this study, the following protocol changes have been implemented at our clinic. The administration interval for the GnRHa trigger was extended by 1 hour, changing from the traditional 36 hours’ interval to 37 hours interval. For the hCG trigger, we maintain the 36 hours interval but will schedule the oocyte retrieval sequence earlier for older patients to prevent delays resulting from the operating room preparation. The outcomes after these changes have been favorable. With an increase in the number of cases, we plan to further evaluate the impact of these adjustments on trigger timing.

This study has several limitations. First, patients with polycystic ovary syndrome or diminished ovarian reserve were not excluded because of the complexity and difficulty of accurately diagnosing these conditions in a large cohort retrospective study. Future research with more comprehensive data will be essential for deriving more accurate conclusions. Second, cases involving dual triggers were excluded because of differences in patient backgrounds compared with those of the GnRHa and hCG groups. Hence, future studies that incorporate dual trigger cases will be necessary to refine the dual trigger protocol. Third, the GnRHa group in this study comprised only cases using buserelin nasal spray administered twice at 1-hour intervals; thus, alternative GnRHa agents, such as leuprorelin injection, may result in different pharmacodynamic effects. Lastly, this study was conducted at a single institution with 99% of participants being Asian, predominantly Japanese. As a result, generalizing the findings to patient populations of other racial backgrounds may be challenging.

## Conclusion

This study examined the impact of the interval from the final ovulation trigger to oocyte retrieval on IVF cycle outcomes. Intervals longer than 36.5 hours led to a higher number of MII oocytes retrieved in the GnRHa trigger group. Conversely, in the hCG trigger group, a higher number of MII oocytes were observed when the interval was shorter than 36.5 hours. The distinction between shorter and longer intervals was more evident among older age groups. Consequently, it is essential to customize protocols on the basis of patient age and trigger type. Additional research incorporating multiple interval thresholds and various trigger types will be needed to identify the best timing for triggering in IVF cycles.

## Declaration of Generative AI and AI-Assisted Technologies in the Writing Process

During the preparation of this work, the authors used Microsoft Copilot (GPT-4.5) to enhance the readability of the English text. After using this tool, the authors meticulously reviewed and edited the content as needed and take full responsibility for the content of the publication. Additionally, we used a professional editor specializing in English academic papers to supervise grammar corrections and provide necessary improvements.

## CRediT Authorship Contribution Statement

**Noritoshi Enatsu:** Writing – original draft, Formal analysis, Data curation. **Kohyu Furuhashi:** Data curation. **Junko Otsuki:** Investigation, Formal analysis, Data curation. **Kunihiro Enatsu:** Writing – review & editing, Investigation. **Eri Okamoto:** Validation, Supervision, Conceptualization. **Shoji Kokeguchi:** Investigation, Data curation, Conceptualization. **Masahide Shiotani:** Supervision, Project administration, Investigation, Formal analysis, Data curation, Conceptualization.

## Declaration of Interests

N. E. has nothing to disclose. K.F. has nothing to disclose. J.O. has nothing to disclose. K.E. has nothing to disclose. E.O. has nothing to disclose. S.K. has nothing to disclose. M.S. has nothing to disclose.
